# Hyperinsulinemia improves ischemic LV function in insulin resistant subjects

**DOI:** 10.1186/1475-2840-9-27

**Published:** 2010-06-24

**Authors:** Patrick M Heck, Stephen P Hoole, Sadia N Khan, David P Dutka

**Affiliations:** 1 Cardiovascular Medicine, ACCI, Level 6, Box 110, Addenbrooke's Hospital, Hills Road, Cambridge, CB2 0QQ, UK; 2 Department of Cardiology, Papworth Hospital, Papworth Everard, Cambridge, CB23 3RE, UK

## Abstract

**Background:**

Glucose is a more efficient substrate for ATP production than free fatty acid (FFA). Insulin resistance (IR) results in higher FFA concentrations and impaired myocardial glucose use, potentially worsening ischemia. We hypothesized that metabolic manipulation with a hyperinsulinemic euglycemic clamp (HEC) would affect a greater improvement in left ventricular (LV) performance during dobutamine stress echo (DSE) in subjects with IR.

**Methods:**

24 subjects with normal LV function and coronary disease (CAD) awaiting revascularization underwent 2 DSEs. Prior to one DSEs they underwent an HEC, where a primed infusion of insulin (rate 43 mU/m ^2^/min) was co-administered with 20% dextrose at variable rates to maintain euglycemia. At steady-state the DSE was performed and images of the LV were acquired with tissue Doppler at each stage for offline analysis. Segmental peak systolic velocities (V_s_) were recorded, as well as LV ejection fraction (EF). Subjects were then divided into two groups based on their insulin sensitivity during the HEC.

**Results:**

HEC changed the metabolic environment, suppressing FFAs and thereby increasing glucose use. This resulted in improved LV performance at peak stress, measured by EF (IS group mean difference 5.3 (95% CI 2.5-8) %, p = 0.002; IR group mean difference 8.7 (95% CI 5.8-11.6) %, p < 0.0001) and peak V _s _in ischemic segments (IS group mean improvement 0.7(95% CI 0.07-1.58) cm/s, p = 0.07; IR group mean improvement 1.0 (95% CI 0.54-1.5) cm/s, p < 0.0001) _, _that was greater in the subjects with IR.

**Conclusions:**

Increased myocardial glucose use induced by HEC improves LV function under stress in subjects with CAD and IR. Cardiac metabolic manipulation in subjects with IR is a promising target for future therapy.

## Background

Insulin increases myocardial glucose utilization and reduces free fatty acid (FFA) oxidation via several mechanisms[1-4]. Glucose oxidation is more oxygen efficient than FFA oxidation[5], hence this change in myocardial metabolism is theoretically beneficial to the heart[5], especially during ischemia, and is thought to be the main mechanism behind the beneficial effects seen in the various trials of glucose-insulin-potassium infusions[6-8].

The presence of insulin resistance (IR) or type 2 diabetes has been shown to affect the metabolic pathway of glucose at multiple levels, from uptake into the myocyte, through to final oxidation within the mitochondria. It has long been known that the subjects with IR are at much greater risk of heart failure and death when compared to insulin sensitive (IS) subjects and these differences cannot be accounted for by other risk factors such as hypertension and vascular disease[9,10].

We hypothesized that altering the myocardial metabolic environment, by creating hyperinsulinemia whilst maintaining euglycemia, would improve ischemic left ventricular function during dobutamine stress in subjects with known coronary artery disease (CAD) and that this effect would be greater in IR subjects.

## Methods

### Subjects

Consecutive subjects with angiographically normal left ventricular (LV) function and symptomatic CAD, awaiting revascularization of stenoses ≥ 75%, were invited to participate. Recruitment was carried out over an 18 month period from a tertiary referral centre for cardiology. Exclusion criteria included LV ejection fraction (EF) of < 40% (either on echocardiography or LV angiography), type 2 diabetes mellitus receiving insulin therapy, significant valvular heart disease, atrial fibrillation, permanent pacemakers or severe co-morbidities. The study had ethical approval from the Local Research Ethics Committee and all subjects gave written informed consent before participating. The study conformed with the principles set out in the Helsinki Declaration.

### Study design

Each subject underwent two dobutamine stress echocardiograms (DSEs) one week apart, thereby acting as their own controls. Both scans were performed before subjects underwent their planned revascularization. One DSE, determined randomly, was performed during the steady-state phase of a hyperinsulinemic, euglycemic clamp (HEC).

### Dobutamine stress echocardiography

Subjects attended for both studies after an overnight fast and their beta-blocker therapy was withheld for 48 hours prior to their attendance. Any oral hypoglycemic agents were stopped 12 hours prior to the studies. After acquiring the resting images, dobutamine was infused intravenously via the antecubital fossa at a starting dose of 10 mcg/kg/min and increased every 3 minutes to doses of 20, 30 and 40 mcg/kg/min. Up to 1.2 mg of atropine, in doses of 300 mcg, was given to subjects who had not reached target heart rate (85% of predicted maximum heart rate) or any other end-point. Subjects underwent continuous 12-lead ECG monitoring, with blood pressure being recorded at baseline and at the end of each stage of dobutamine.

Standard clinical endpoints were used, including intolerable symptoms (such as chest pain), > 2 mm of planar or down-sloping ST-segment depression, persistent arrhythmias such as ventricular tachycardia or atrial fibrillation, and hypotension (a fall of systolic pressure 30 mmHg) or bradycardia.

#### Image acquisition

All echocardiographic images were obtained with a standard, 2D echocardiography system (Vivid Seven, GE Medical Systems) and stored digitally. Standard apical views of the LV were acquired at rest and at each stage of dobutamine stress with the patient in the left recumbent position. To minimize beat-to-beat variability, all recordings were made in gently held end-expiration and stored for subsequent off-line analysis (Echo PAC PC software, GE Medical Systems).

#### Analysis

An expert observer blinded to the clinical data and tissue Doppler results performed all the echocardiographic analyses. The ejection fraction (EF), left ventricular end-diastolic volume (LVEDV) and end-systolic volume (LVESV) were calculated from the apical views using the modified Simpson's biplane method[11]. From these measurements, stroke volume (SV) and cardiac output (CO) were then also calculated.

### Tissue Doppler imaging

#### Acquisition

Color tissue Doppler imaging (TDI) was used to assess longitudinal myocardial tissue velocities and myocardial strain generation at baseline and each stage of dobutamine stress. The filter was set to exclude high frequency signal, and the Nyquist limit was set to 24 cm/s to minimize aliasing during stress. The sector angle and imaging depth were minimized to maintain adequate frame rates of at least 140 frames per second.

#### Analysis

Off-line analysis was performed by one observer, blinded to the nature of the study. A 6 × 6 mm sampling area was applied to the annulus, basal and mid segments of each wall of the LV. Apical segments were not analyzed for velocity because off-line velocities in these segments have been shown to be unreliable[12]. Analysis of myocardial strain was performed on all 16 LV segments. A 6 by 12 mm sample area was placed in the middle of each segment. It was then tracked semi-automatically throughout each cardiac cycle to ensure that it remained in the middle of the segment and did not sample the LV blood pool. Peak systolic (V_s_) velocity and post-systolic strain (PSS) were recorded from 3 beats and averaged for each sample point. These measurements were chosen as they have been shown to be the more sensitive and reproducible TDI markers of ischemia in DSE[12-16]. The intraobserver and interobserver variability for TDI measurements in our intuition are 11% and 17% respectively.

The LV segments were classified as either 'ischemic' or 'non-ischemic' on the basis of the anatomy of the subject's coronary artery disease and the predicted arterial blood supply to each segment, based on the guidelines from the ASE[17].

### Hyperinsulinemic, euglycemic clamp

A hyperinsulinemic euglycemic clamp was performed as described previously[18]. After patients had fasted overnight, a cannula was inserted into a vein in the antecubital fossa for infusion of glucose and insulin. A second cannula was inserted in the opposite arm, which was arterialized using a heating pad set at 50°C. After an initial loading dose, insulin (Human Actrapid, Novo Nordisk) was infused at a constant rate of 43 mU/min/m ^2 ^of body surface area. Blood glucose levels were sampled every 5 to 10 minutes from the opposite cannula and analyzed using the glucose oxidase method (YSI 2300, YSI Life Sciences, Ohio). Glucose infusion rates were adjusted accordingly, with the aim of maintaining constant glucose levels[18]. Once steady state was achieved, as indicated by 3 consecutive blood glucose values that were within 5% of one another, the subjects underwent DSE as described above. The insulin infusion was stopped at the end of the peak dobutamine stress stage, along with the dobutamine infusion, but the glucose continued for an additional 20 minutes to avoid rebound hypoglycemia.

### Assessment of insulin resistance

The glucose clamp technique, originally developed by DeFronzo et al.[18], is widely accepted as the reference standard for directly determining metabolic insulin sensitivity in humans[19]. After at least one hour of constant insulin infusion, steady-state conditions can typically be achieved for plasma insulin, blood glucose and the glucose infusion rate. It is assumed that the hyperinsulinemic state is sufficient to completely suppress hepatic glucose production. Accordingly, since there is no net change in blood glucose concentrations under steady-state clamp conditions, the glucose infusion rate must be equal to the glucose disposal rate, and this value is termed M-value. Thus, whole body glucose disposal at a given level of hyperinsulinemia can be determined directly. M-value is typically normalized to body weight or fat-free mass to generate an estimate of insulin sensitivity (mg/kg/min).

#### Biochemical analysis

In addition to blood glucose sampling, serum insulin, c-peptide (both using commercially available AutoDELFIA Automatic Immunoassay kits, PerkinElmer, Waltham, MA) and FFA (modified Roche Free Fatty Acid enzymic colorimetric kit assay) concentrations were measured at baseline, steady state (pre-DSE), peak stress and 30 minutes into recovery during the clamped study and also at baseline, peak stress and 30 minutes into recovery of the control (unclamped) study.

The coefficients of variation for the insulin assay ranges from 3.1% at 29 pmol/l to 1.9% at 277 pmol/l. For the c-peptide assay: 4.8% at 472 pmol/l to 3.7% at 2056 pmol/l and for the FFA assay: 10.6% at 112 μmol/l and 4.3% at 465 μmol/l.

#### Statistical analysis

The primary outcome for the study was LV EF. The study had a 90% power to detect an absolute improvement in EF of 5% in the clamp versus the control group with a significance level of 0.05. The analyses were restricted to the 24 patients who had both control and clamped studies. Descriptive summaries are expressed as the mean (standard deviations) for continuous measurements and as the number (percentage) for categories. Comparisons between control and clamped measurements were made using paired Student's t-tests. Comparison between different stages of the clamp were done by the ANOVA method. Fisher's exact test was used to compare the incidence of PSS in the control and clamp study. The statistical software programs SPSS 12.0.1 (SPSS, Cary, NC) was used for the statistical analyses.

## Results

Twenty-six subjects were recruited of whom 24 completed the study. One subject was unable to complete the study due to emergency admission with unstable angina, and one subject withdrew consent. The median M-value for the 24 subjects was 3.2 mg/kg/min. The subjects were thus divided into two groups; those with an M-value less than 3.2 mg/kg/min (the insulin resistant (IR) group) and those with an M-value greater then 3.2 mg/kg/min (the insulin sensitive (IS) group). The demographics for the two groups are shown in Table 1. There were no differences between the two groups except for weight.

**Table 1:** Demographics of IS and IR groups

Parameters	Insulin Sensitive (IS)	Insulin Resistant (IR)	P
Age	64 (8)	64.2 (8)	ns
Male, *n *(%)	11 (92)	10 (83)	ns
			
**Medication, *n *(%)**			
β-Blocker	11 (92)	11 (92)	ns
Nitrate	9 (75)	6 (50)	ns
Calcium Channel Blocker	2 (17)	3 (25)	ns
Statin	12 (100)	11 (92)	ns
Ejection Fraction, %	62.2 (4)	63.3 (6)	ns
**Coronary Disease, *n *(%)**			
LAD stenosis	6 (50)	10 (83)	ns
LCx stenosis	4 (33)	3 (25)	ns
RCA stenosis	5 (42)	5 (42)	ns
Single vessel CAD	10 (83)	8 (67)	ns
Two vessel CAD	1 (8)	2 (17)	ns
Three vessel CAD	1 (8)	2 (17)	ns
			
Weight (Kg)	82 (15)	97.5 (20)	0.04
BSA (m^2^)	1.96 (0.2)	2.1 (0.2)	ns
M Value (mg/kg/min)	5.7 (1.3)	2.2 (0.7)	< 0.0001
Diabetes* *n *(%)	3 (25)	6 (50)	ns
Diet controlled	3 (25)	2 (17)	ns
Metformin	0	3 (25)	ns
Metformin and sulphonyl urea	0	1 (25)	ns

BSA = body surface area. * = subjects with a clinical diagnosis of type 2 diabetes receiving oral hypoglycemic agents. Ns = not significant

### Dobutamine stress

The dose of dobutamine and atropine administered in each group were similar. This equivalent level of pharmacological stress resulted in heart rates (HRs), systolic blood pressures (SBPs) and rate pressure products (RPPs) that did not differ significantly between the control and clamped scans at peak dobutamine stress in either the IS or IR group (Table 2). There were no differences between the IS and IR groups in the hemodynamic response shown with dobutamine stress in both control and clamped studies.

**Table 2:** Peak HR, blood pressure and RPP during DSE in IS and IR groups

		Insulin Sensitive			Insulin Resistant		
		Control	Clamp	p	Control	Clamp	p
Max Dobutamine dose (μg/kg/min)		37.5 (4.5)	38.3 (3.9)	ns	35.8 (5.1)	35 (5.2)	ns
Mean Atropine dose (μg)		150 (239)	150 (239)		100 (233)	100 (233)	
**Hemodynamic variables**							
Mean %max HR achieved		83 (8)	83 (5)	ns	81 (7)	80 (9)	ns
**Peak**	HR (bpm)	130 (15)	130 (10)	ns	126 (10)	124 (13)	ns
	SBP (mmHg)	147 (21)	143 (20)	ns	142 (25)	141 (25)	ns
	RPP (bpm.mmHg)	19026 (2850)	18554 (2925)	ns	17897 (3298)	17540 (3979)	ns

Ns = not significant

### Biochemical analyses

**Figure 1:**
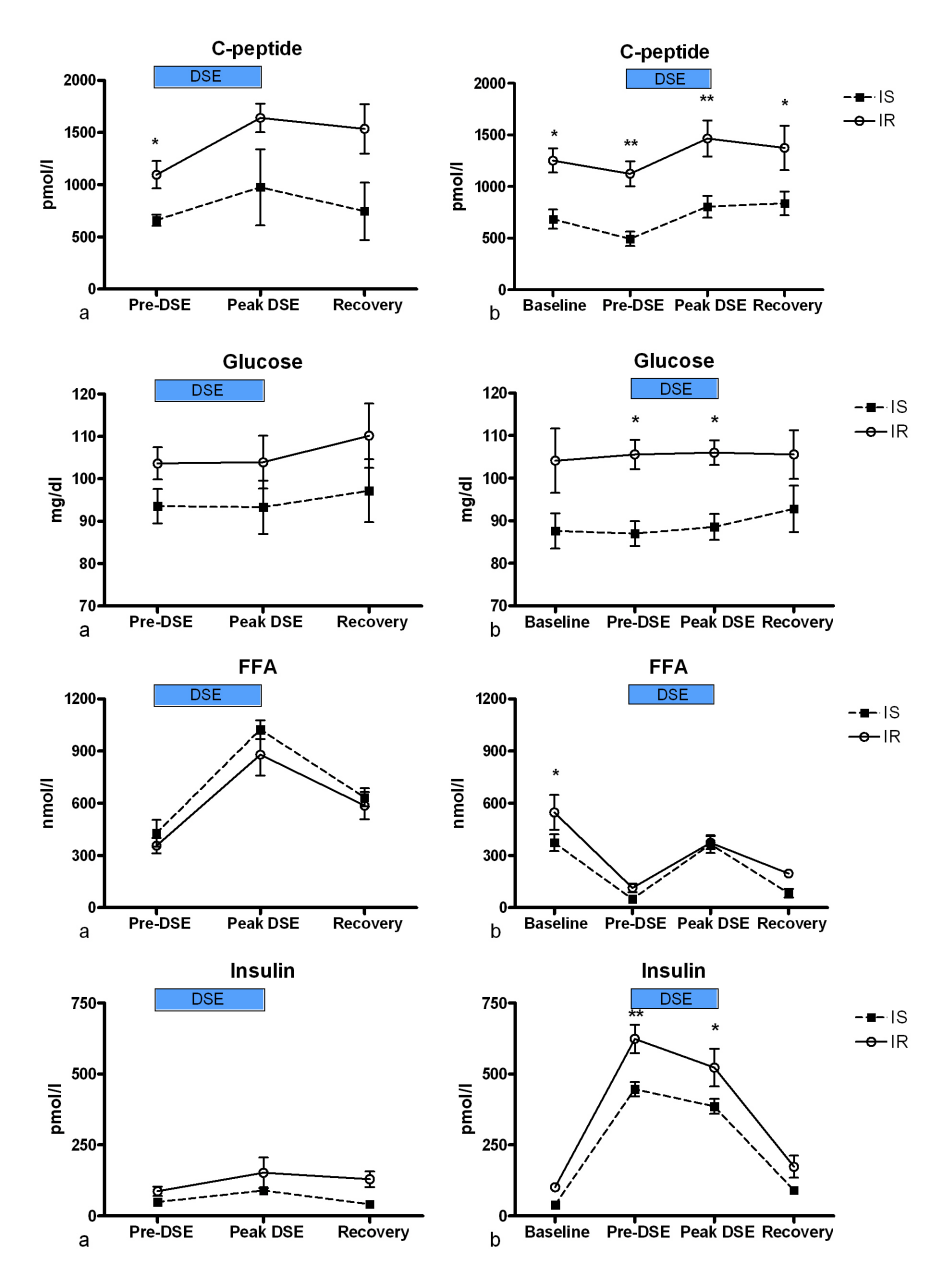
**Metabolic parameters during control and clamped studies**. shows the glucose, c-peptide, FFA and insulin concentrations during control, graphs (a), left, and clamped studies, graphs (b), right. The black square represents the IS group and the white circle the IR group. Numbers plotted are mean values and the standard errors. Detailed description of the results is in the main text. * = p < 0.05; ** = p < 0.01 for comparison between IS and IR group.

The metabolic responses of both groups during the clamp are shown in Table 3 and Figure 1.

**Table 3:** Metabolic parameters for clamp studies

Glucose (mg/dl)	Insulin Sensitive	Insulin Resistant	p	C-peptide (pmol/l)	Insulin Sensitive	Insulin Resistant	p
**Baseline**	88 (14)	104 (27)	0.07	**Baseline**	683 (291)	1251 (352)	0.001
**Steady state**	86 (11)	106 (13)	0.0004	**Steady state**	493 (219)	1124 (361)	0.0002
**Peak Stress**	88 (11)	106 (11)	0.0004	**Peak Stress**	804 (334)	1465 (524)	0.004
**Recovery**	93 (18)	106 (20)	ns	**Recovery**	836 (363)	1374 (646)	0.04
							

**Insulin (pmol/l)**				**FFA (nmol/l)**			

**Baseline**	38.9 (18.7)	102 (27.6)	< 0.0001	**Baseline**	374 (153)	548 (299)	ns
**Steady state**	447 (78.6)	624 (150)	0.004	**Steady state**	49.2 (51.3)	114 (72)	0.04
**Peak Stress**	386 (82.9)	523 (198)	0.06	**Peak Stress**	365 (160)	374 (113)	ns
**Recovery**	90.1 (45.3)	174 (117)	0.05	**Recovery**	83.9 (79.1)	197 (52.1)	0.002

FFA = free fatty acids. Ns = not significant

#### C-Peptide

The c-peptide concentrations were significantly higher in the IR groups at every stage of the clamp.

#### Glucose

The baseline glucose concentration in the IS group was lower than in the IR group (88 (14) vs. 104 (27) mg/l, p = 0.07), although this did not achieve statistical significance. At both the steady state and peak DSE stages of the clamp, the difference in the glucose concentration in the IS group did achieve statistical significance.

#### Insulin

Baseline fasted insulin concentration in the IS group was significantly lower than the IR group (38.9 (18.7) vs. 102 (27.6) pmol/l, p < 0.0001). Although there is considerable overlap in insulin concentrations between normal and insulin resistance, a fasting serum insulin concentration of greater than the upper limit of normal for the assay used (approximately 60 pmol/l) has been considered a marker of insulin resistance[20,21] and this supports the separation of the subjects into IS and IR groups using M value. This trend in lower insulin concentration in the IS groups was present at every stage of the clamp, although it did not achieve statistical significance at the peak DSE or recovery stages.

#### FFA

The HEC resulted in more marked suppression of free fatty acid production in the IS group, resulting in a significantly lower FFA concentration at steady state. This difference was negated by the profound lipolytic effect of dobutamine at peak stress, but was again present in the recovery stage.

### 2D echo measurements

The LV volumes, EF and CO are shown in Table 4. Measurements were made just prior to commencing the dobutamine infusion ('Pre-DSE'), at peak dobutamine stress ('Peak') and 15 minutes after stopping the dobutamine infusion ('Recovery').

**Table 4:** 2D echocardiographic measurements

Echocardiographic variables		Insulin Sensitive					Insulin Resistant				
		Control		Clamp		p	Control		Clamp		p
**Pre-DSE**	EDV (ml)	102	(16)	99.2	(19)	ns	108	(24)	103	(27)	ns
	ESV (ml)	38.6	(7)	36	(9)	ns	40.1	(13)	37.5	(13)	ns
	SV (ml)	63.6	(11)	63.2	(13)	ns	68	(15)	65.4	(15)	ns
	CO (L/min)	3.7	(0.4)	3.6	(.7)	ns	4.2	(1.1)	4	(1.1)	ns
	EF (%)	62.2	(4)	63.8	(4.5)	ns	63.3	(6)	64.3	(5.9)	ns
**Peak**	EDV (ml)	74.9	(14)	74.8	(16)	ns	86.1	(22)	93.3	(28)	ns
	ESV (ml)	26.4	(9)	22.1	(7)	0.09	31.3	(14)	25.4	(11)	0.03
	SV (ml)	48.5	(8)	52.6	(12)	ns	54.8	(11)	68	(19)	0.003
	CO (L/min)	6.3	(1.4)	6.8	(1.7)	ns	6.9	(1.5)	8.3	(2.1)	0.001
	EF (%)	65.2	(6.1)	70.4	(6.3)	0.002	64.8	(7.2)	73.5	(5.8)	< 0.0001
**Recovery**	EDV (ml)	92.2	(21)	98.7	(21)	ns	103	(24)	104	(23)	ns
	ESV (ml)	35.5	(11)	39	(11)	ns	40.5	(15)	35.8	(13)	0.09
	SV (ml)	56.6	(13)	58.7	(12)	ns	62	(12)	68	(12)	0.05
	CO (L/min)	4.4	(0.8)	4.7	(0.8)	ns	4.7	(0.8)	5	(1)	ns
	EF (%)	61.8	(5.9)	60.4	(4.5)	ns	61.5	(7.5)	66.4	(6.7)	0.03

Ns = not significant

#### Ejection fraction

The pre-DSE EF were unaffected by the clamp in both groups. At peak stress the clamp significantly improved the EF in both the IS group mean improvement 5.3% (95% CI 2.5 to 8), p = 0.002; IR group mean difference 8.7% (95% CI 5.8 to 11.6), p < 0.0001), with a larger magnitude effect in the IR group. At the recovery stage, the clamp continued to have a positive effect on the IR group (mean improvement 4.8% (95% CI, 0.7 to 8.9), p = 0.03), but not in the IS group (mean improvement 1.4% (95% CI, -2.1 to 5), p = 0.4).

#### EDV

There was no significant effect of HEC on EDV in either IS or IR groups at any stage.

#### ESV

At peak dobutamine stress the ESV was significantly reduced in the clamp study in the IR group (mean difference 5.9 (5.3) mls, p = 0.03). There was a reduction in the ESV of the IS group at peak stress, but the magnitude of effect was smaller than in the IR group and it did not achieve statistical significance (mean difference 4.3 (5) mls, p = 0.09).

#### SV and CO

At the pre-DSE stage the clamp exerted no effect on either SV or CO in either group. At peak stress both SV and CO were significantly improved in the IR group (SV mean difference 13.1 (8) mls, p = 0.003; CO mean difference 1.4 (0.7) l/min, p = 0.001), but not in the IS group (SV mean difference 4.2 (7) mls, p = 0.2; CO mean difference 0.5 (1) l/min, p = 0.3). There were no significant differences in the SV and CO in the recovery stage.

### Tissue Doppler imaging

#### Peak dobutamine V_s_

The results for the peak V_s _are shown in Table 5. Paired tissue Doppler systolic velocities (V_s_) were recordable in 94.4% (272 out of 288) at peak dobutamine. The V_s _velocities were consistently higher for all segments in the clamped studies, but it was only in the IR group that this improvement achieved statistical significance, and it was most significant in the segments classified as ischemic (IS ischemic segments mean improvement 0.7(95% CI 0.07-1.58) cm/s, p = 0.07; IR ischemic segments mean improvement 1.0 (95% CI 0.54-1.5) cm/s, p < 0.0001).

**Table 5:** Mean segmental V_s _at peak dobutamine stress

	Insulin Sensitive				Insulin Resistant			
	Control	Clamp	*n*	p	Control	Clamp	*n*	p
**Annulus points**	10.6 (2.5)	10.9 (2.3)	72	ns	9.0 (3.3)	10.0 (3.0)	66	0.002
**Ischemic segments**	8.6 (4.0)	9.3 (4.2)	57	0.07	5.9 (3.1)	6.7 (2.8)	66	< 0.0001
**Non-ischemic seg.**	8.4 (3.4)	8.5 (3.0)	82	ns	7.3 (3.4)	8.3 (3.6)	67	0.02

*n *= number of paired values used for analysis. Ns = not significant.

#### Post systolic strain (PSS)

The incidences of PSS at baseline and the development of new PSS at peak stress and recovery are shown in Table 6. The incidence of PSS at baseline was similar in each group are compares well to the reported incidence of PSS in normal myocardium[14]. At peak DSE and in recovery in both groups the clamp resulted in fewer segments exhibiting new PSS (i.e. segments that did not have PSS at baseline), but this effect was most marked, and achieved statistical significance, in the recovery stage in the IR subjects (15 segments in the clamp vs. 30 in the control, p = 0.03), suggesting less post-ischemic dysfunction.

**Table 6:** Number of myocardial segments with post systolic strain (PSS)

	Insulin Sensitive			Insulin Resistant		
Baseline PSS	Control	Clamp	p	Control	Clamp	p
Ischemic	21 (25)	27 (32)	ns	36 (31)	44 (38)	ns
Non-ischemic	30 (28)	31 (29)	ns	24 (32)	22 (30)	ns
**New PSS at Peak Stress**						
Ischemic	31 (48)	26 (45)	ns	40 (49)	34 (47)	ns
Non-ischemic	40 (52)	30 (39)	ns	20 (39)	22 (42)	ns
**New PSS in Recovery**						
Ischemic	21 (33)	16 (28)	ns	30 (37)	15 (21)	0.03
Non-ischemic	29 (38)	25 (33)	ns	11 (22)	6 (12)	ns

Data shown is n (%). Ns = not significant.

## Discussion

This study shows that in subjects with flow-limiting CAD alteration of myocardial substrate utilization improves LV function during DSE in both IR and IS subjects and lessens post-ischemic dysfunction in IR subjects. Whilst similar effects were seen in both groups, the magnitude was less in the IS group and so it did not generally achieve statistical significance in this study. Overall, though, the effects are fairly modest and whilst the sample size is small, the improvements observed in the clamped DSE in IR group, in particular the reduction in post-ischemic dysfunction are likely to have clinical significance.

### Left ventricular performance during stress

Euglycemic hyperglycemia during DSE significantly improved the primary endpoint of LV EF at peak stress in both IS and IR groups. Whilst the LV EF was significantly improved by the HEC in the IS group, it was the only one of the indices of LV function that was significantly improved. Peak stress SV, ESV, CO and V_s _were slightly increased in the IS group, but not significantly so. Whereas, in the IR group at peak stress SV, ESV, CO and V_s _were all significantly improved.

The improvements in the IR group were not limited to the peak systolic function. Post-systolic thickening or strain is a well reported and sensitive marker of stunning[22-27]. In the IS group the clamp tended to result in fewer segments with PSS in the recovery stage, but the difference was not statistically significant. In the IR group, however, the effect was far more pronounced and statistically significant, with almost half as many segments exhibiting PPS in the recovery stage of the clamp compared to the control.

### Metabolic environment

As expected, baseline metabolic profiles of the two groups were different, with the IR group having higher insulin and c-peptide concentrations. The FFA and glucose concentrations were also elevated in the IR group, but not significantly so, due in part to the sub-study being relatively underpowered to assess these. These differences are expected, as the groups were not randomly selected, but selected on the basis of each subject's insulin sensitivity, measured during the HEC. Elevated fasting glucose, insulin and FFA concentrations are all part of the phenotype seen in insulin resistance[20,28].

At the steady state phase of the clamp, before the start of the dobutamine stress, the differences in the metabolic measurements persisted. Despite the higher insulin concentration in the IR group, lipolysis inhibition was not as complete, with a persisting elevation of the FFA concentration compared to the IS group. As one of the main driving forces for myocardial FFA uptake and utilization is substrate availability[29,30], it would therefore be expected that the myocardium in the IR groups will be using more FFAs than in the IS group and that this might disadvantage the IR group.

### Possible mechanisms

Whilst the metabolic environment just prior to commencing the dobutamine stress may not have been as well optimized in the IR group as the IS group, with presumed higher FFA use and lower glucose use, it is apparent that the improvement in LV performance during DSE seen with the clamp in the IR group was greater than that observed in the IS group. There are several possible reasons to explain this, of which all or none may contribute.

The normal myocardial response to ischemia is to increase glucose uptake, glycogenolysis and glycolysis[31], as well as increase FFA oxidation[32]. Perhaps, in insulin sensitive individuals, challenged with only modest amounts of myocardial ischemia as in this study, this intrinsic metabolic switch is adequate and additional stimulation of glucose oxidation with the HEC provides minimal incremental benefit in this group, whereas in the IR group this intrinsic metabolic switching mechanism is impaired but can be overcome with the HEC.

Also, insulin resistance has been shown to have several adverse effects on the metabolism of the myocardium that heighten the effects of ischemia. These include reduced glucose uptake[33-35] and oxidation[36], increased FFA uptake and oxidation[37,38] as well as decrease in calcium transport within the sarcolemma and alterations in myofibrillary regulatory contractile proteins[39]. The net effect is a reduction in cardiac efficiency[40] at rest that may predispose to diabetic cardiomyopathy[41-43], but that also causes increases the susceptibility of the insulin resistant heart to myocardial ischemia and to a greater reduction in myocardial performance for a given amount of ischemia compared with the normal heart[44-46]. It therefore stands to reason that insulin resistant myocardium has far more to gain by optimization of its metabolism, so provided the hyperinsulinemia generated by the clamp is sufficient to overcome the IR, then the improvement in myocardial performance during and after ischemia will be greater than in IS subjects.

An alternative explanation is the improved LV performance observed in the IR group may be accounted for by the elevated glucose and insulin concentration present throughout the clamp stimulating greater myocardial glucose uptake, compared to the IS group. However, work by others has shown that subjects with IR can have impaired glucose uptake compared to IS subjects, even during hyperinsulinemia[47,48], making the additional hyperinsulinemia seen in the IR subjects in this study unlikely to result in significantly greater myocardial glucose uptake compared to the IS subjects, although this cannot be proved.

### Study limitations

The most significant limitation of this study is the sample size. The study was powered to assess EF, which was improved in both groups, and it may have been underpowered to assess some of the other variables. It is possible, therefore, that some of the TDI variables that were not significantly different in the IS group, but showed trends of improvement with the clamp, failed to achieve significance due to a lack of power.

Another limitation of this study is use of quantative QCA to define ischemic myocardial segments as opposed to functional imaging such as myocardial perfusion SPECT. Subjects will have variable coronary anatomy and functional imaging would be better as assessing which segments were ischemic.

Also, a number of assumptions on myocardial metabolism and the effect the HEC has on this have had to be made. Direct measurement of myocardial substrate metabolism is challenging, requiring a combination of arterial and coronary sinus venous blood sampling or sophisticated PET imaging with radio-labeled substrates such as glucose and palmitate[49,50]. However, previously-reported work by others has investigated the effects of HEC on myocardial metabolism in humans[51,52], so the assumptions are evidence-based.

## Conclusions

This study shows that hyperinsulinemic euglycemia during DSE improves LV function in both IR and IS subjects with flow-limiting coronary disease. It also suggests that subjects with IR may benefit more in terms of increased cardiac performance and reduced post-ischemic dysfunction compared insulin sensitive subjects.

## Competing interests

The authors declare that they have no competing interests.

## Authors' contributions

All authors have read and approved the final manuscript. PMH participated in the project design and subject enrollment. He also performed the DSE studies and clamps, performed the analyses and wrote the manuscript. SPH participated in subject enrollment, aided in the DSE studies and clamps and reviewed the manuscript. SNK participated in the project design, subject enrollment and DSE analysis, as well as reviewing the final manuscript. DPD designed the project, participated in the results analysis and reviewed the manuscript.
